# Programmed Protection of Foreign DNA from Restriction Allows Pathogenicity Island Exchange during Pneumococcal Transformation

**DOI:** 10.1371/journal.ppat.1003178

**Published:** 2013-02-14

**Authors:** Calum Johnston, Bernard Martin, Chantal Granadel, Patrice Polard, Jean-Pierre Claverys

**Affiliations:** 1 Centre National de la Recherche Scientifique, LMGM-UMR5100, Toulouse, France; 2 Université de Toulouse, UPS, Laboratoire de Microbiologie et Génétique Moléculaires, Toulouse, France; Harvard Medical School, United States of America

## Abstract

In bacteria, transformation and restriction-modification (R-M) systems play potentially antagonistic roles. While the former, proposed as a form of sexuality, relies on internalized foreign DNA to create genetic diversity, the latter degrade foreign DNA to protect from bacteriophage attack. The human pathogen *Streptococcus pneumoniae* is transformable and possesses either of two R-M systems, DpnI and DpnII, which respectively restrict methylated or unmethylated double-stranded (ds) DNA. *S. pneumoniae* DpnII strains possess DpnM, which methylates dsDNA to protect it from *Dpn*II restriction, and a second methylase, DpnA, which is induced during competence for genetic transformation and is unusual in that it methylates single-stranded (ss) DNA. DpnA was tentatively ascribed the role of protecting internalized plasmids from *Dpn*II restriction, but this seems unlikely in light of recent results establishing that pneumococcal transformation was not evolved to favor plasmid exchange. Here we validate an alternative hypothesis, showing that DpnA plays a crucial role in the protection of internalized foreign DNA, enabling exchange of pathogenicity islands and more generally of variable regions between pneumococcal isolates. We show that transformation of a 21.7 kb heterologous region is reduced by more than 4 logs in *dpnA* mutant cells and provide evidence that the specific induction of *dpnA* during competence is critical for full protection. We suggest that the integration of a restrictase/ssDNA-methylase couplet into the competence regulon maintains protection from bacteriophage attack whilst simultaneously enabling exchange of pathogenicicy islands. This protective role of DpnA is likely to be of particular importance for pneumococcal virulence by allowing free variation of capsule serotype in DpnII strains via integration of DpnI capsule loci, contributing to the documented escape of pneumococci from capsule-based vaccines. Generally, this finding is the first evidence for a mechanism that actively promotes genetic diversity of *S. pneumoniae* through programmed protection and incorporation of foreign DNA.

## Author Summary

Natural genetic transformation can compensate for the absence of sexual reproduction in bacteria, allowing genetic diversification by recombination. It proceeds through the internalization of single stranded (ss) DNA fragments created from an exogenous double stranded (ds) DNA substrate, which are incorporated into the genome by homology. On the other hand, restriction-modification (R-M) systems, which protect bacteria from bacteriophage attack by degrading invading foreign DNA, potentially antagonize transformation. About half of the strains of the naturally transformable species and human pathogen *Streptococcus pneumoniae* possess an R-M system, DpnII, restricting unmethylated dsDNA. DpnII strains possess DpnA which is unusual in that it methylates ssDNA. Here we show that DpnA plays a crucial role in the protection of internalized heterologous transforming ssDNA, preventing the post-replicative destruction by DpnII of transformants produced by chromosomal integration of heterogolous DNA by virtue of flanking homology. This protective role of DpnA is of particular importance for acquisition of pathogenicity islands, such as capsule loci, from non-DpnII origin by DpnII strains, likely contributing to pneumococcal virulence via escape from capsule-based vaccines. Generally, this finding is the first evidence for a mechanism that actively promotes genetic diversity of *S. pneumoniae* through active protection and incorporation of foreign DNA.

## Introduction

While sexual reproduction, which is crucial for genetic diversity in eukaryotes, is lacking in bacteria, genetic transformation is regarded as a substitute [1]. Genetic transformation proceeds through the internalization of single stranded (ss) DNA fragments created from an exogenous double stranded (ds) DNA substrate, which are incorporated into the genome by homology. This forms a heteroduplex of one strand of host DNA associated with complementary exogenous ssDNA, which is resolved by replication, producing one wild-type daughter chromosome, and one possessing the mutation originally present on the exogenous DNA. This widespread process [2] contributes to genetic plasticity of the major human pathogen *Streptococcus pneumoniae* (the pneumococcus) [3], potentially leading to antibiotic resistance acquisition and vaccine escape [4]. Current pneumococcal vaccines target the polysaccharide capsule, which is considered the main pneumococcal virulence factor, and of which over 90 different serotypes exist [5]. Cumulatively, these serotype loci are almost equivalent in size to a single pneumococcal genome, demonstrating the high levels of genetic diversity present in the pneumococcal population. Vaccine escape presumably occurs via exchange of capsule loci, ranging in size from 10.3 kb to 30.3 kb, by transformation. These loci occupy the same position in the genome, between the *dexB* and *aliA* genes which provide flanking homology for chromosomal integration of heterologous capsule sequences.

**Figure 1:**
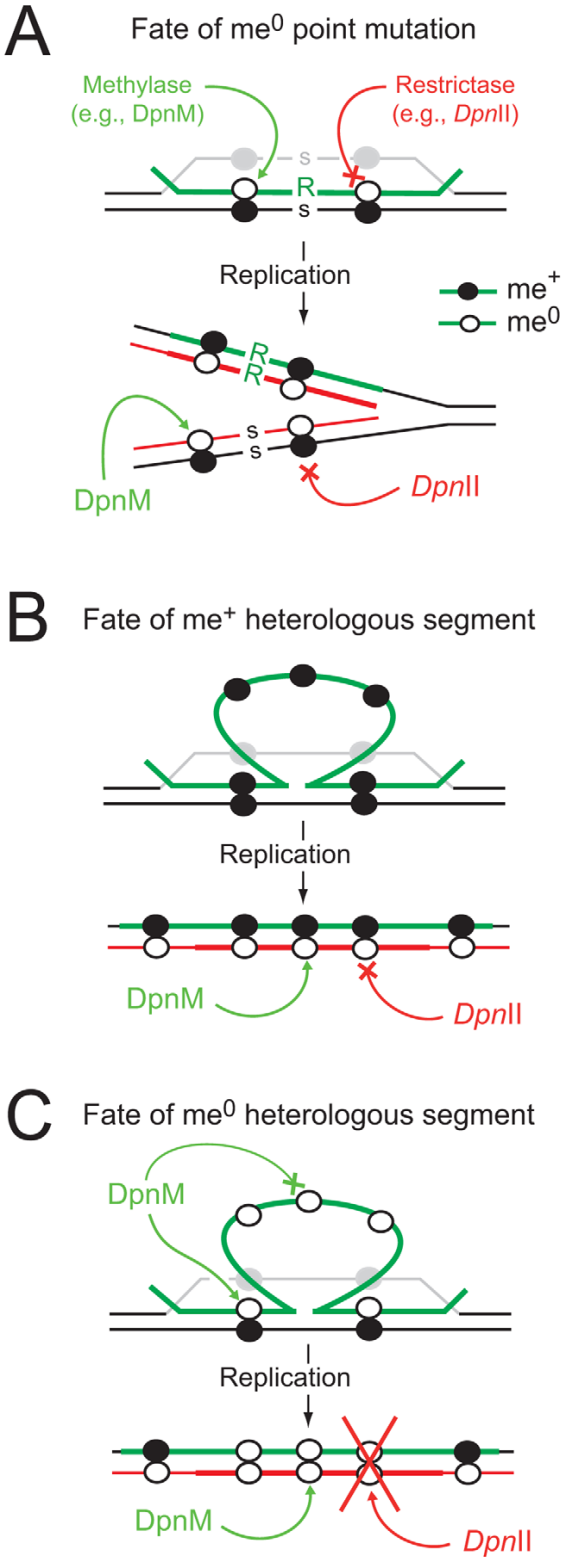
Diagrammatic representation of the impact of R-M systems on transformation. (A) Integration of a point mutation carried by me^0^ donor DNA is not affected by restriction. Color code: green, transforming (donor) DNA; black, host DNA; grey, displaced recipient strand; red, newly replicated DNA. me^+^ DNA shown as closed circles and me^0^ DNA as open circles; point mutation as R in donor and s for recipient. (B) Integrated me^+^ heterologous donor DNA is resistant to *Dpn*II, which is unable to cleave me^+/0^ dsDNA produced by replication. Color code as above; thick red, neosynthesized complement to heterology. (C) Integrated me^0^ heterologous donor DNA is sensitive to restrictase (e.g., *Dpn*II) because me^0^ dsDNA is produced by replication. We hypothesize that DpnA, which, unlike DpnM, acts on ssDNA, protects the transformation intermediate by ensuring production of me^+/0^ dsDNA after replication (as in panel B).

On the other hand, many bacteria possess DNA restriction-modification (R-M) systems, which defend against invasion by foreign DNA such as bacteriophage, and are seen as restrictive to genetic diversity, potentially antagonizing genetic exchange processes. R-M systems classically encode a restrictase, which degrades unmethylated (me^0^) foreign dsDNA, and a methylase which methylates the dsDNA of the host genome, protecting it from the restrictase [6]. In the course of transformation and despite the widely accepted notion that R-M systems antagonize genetic exchange processes, internalized me^0^ ssDNA is resistant to restriction as most restrictases cannot cut ssDNA. Upon transfer of a point mutation, heteroduplex formation produces hemimethylated (me^+/0^) dsDNA, which remains resistant to restriction, but can be methylated by the dsDNA methylase (Fig. 1A). Resolution of the heteroduplex by replication then forms two me^+/0^ dsDNA, which again remain resistant to restriction. As a result, at no point during the transfer of a point mutation is the internalized or chromosomally-integrated DNA sensitive to restriction. The situation is potentially different for the transfer of heterologous DNA, since the heterologous region remains in the form of a ss loop after heteroduplex formation, flanked by regions of homology (Fig. 1BC). In the case of methylated (me^+^) transforming DNA, this should not be problematic, as the integrated ssDNA is already methylated, confering resistance to restriction to the dsDNA formed at the region of heterology after resolution of the heteroduplex by replication (Fig. 1B). However, in the case of me^0^ transforming DNA, resolution of the heteroduplex by replication creates me^0^ dsDNA, which should be sensitive to restriction (Fig. 1C). As a result, classical R-M systems should severely limit the acquisition of heterologous sequences of me^0^ origin.

**Figure 2:**
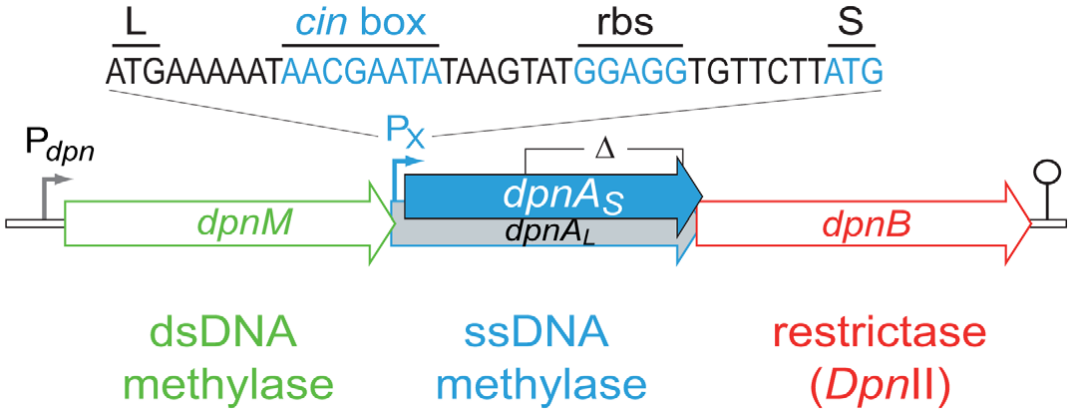
Organization of the *dpnII* locus. Schematic representation of the locus in *S. pneumoniae*, with the identity of the competence-induced promoter and DpnA start codons indicated. Open circle, terminator. P*_dpn_* and P_X_, σ^70^ and σ^X^ dependent promoters, respectively; L and S, start codon of *dpnA_L_* and *dpnA_S_*, respectively; *cin* box, σ^X^ binding site; rbs, ribosome-binding site. The limits of the deletion internal to *dpnA* (Δ) previously constructed [13] and used in this study are indicated.

*S. pneumoniae* strains possess one of two complementary R-M systems, DpnI or DpnII [7], encoded at a common location in the chromosome [8]. *Dpn*I is an atypical restriction enzyme, as it cleaves me^+^ dsDNA. Chromosomal DNA produced by replication in DpnI cells is me^0^ and therefore is not sensitive to *Dpn*I restriction. In contrast to this, DpnII strains possess a classical companion dsDNA methylase (DpnM), which methylates host DNA after replication, rendering the chromosome resistant to the *Dpn*II restrictase (encoded by *dpnB*), while *Dpn*II protects the cell from me^0^ bacteriophage attack by restricting me^0^ dsDNA. The *dpnII* locus also encodes DpnA (Fig. 2), a methylase with the unusual ability to specifically modify ssDNA (ss-methylase). Whilst the locus is under the control of the constitutive P*_dpn_* promoter, *dpnA* is also specifically induced from P_X_, a competence-inducible (*cin*) promoter recognized by the competence-specific σ factor, ComX [9],[10]. This alternative σ is transiently active when pneumococcal cells become competent for genetic transformation [11] and required for synthesis and assembly of a dedicated complex for uptake and integration of exogenous DNA into the chromosome, known as the transformasome [12].

The primary role of DpnA was suggested to be protection of plasmids taken up by genetic transformation from *Dpn*II restriction, via methylation of internalized plasmid ssDNA fragments [13],[11]. Authors demonstrated that installation of me^0^ plasmids in *S. pneumoniae* was drastically reduced in a DpnII strain lacking DpnA, and concluded that DpnA biological role was to protect me^0^ plasmids from *Dpn*II [13]. However, this appeared unlikely to be the function of DpnA, since plasmids naturally carried by *S. pneumoniae* are extremely rare [14],[15] and plasmid transformation is strikingly inefficient in this species [16]. An alternative hypothesis was therefore proposed, that the role of DpnA was to protect heterologous me^0^ ssDNA to allow exchange of pathogenicity islands and, more generally, plasticity islands, defined as chromosomal regions variable between pneumococcal isolates [3]. According to this hypothesis, DpnA should methylate internalized transforming me^0^ ssDNA. Resolution of the transformation heteroduplex by replication should then result in formation of me^+/0^ rather than me^0^ dsDNA, thus protecting the transformed chromosome from restriction by *Dpn*II. Thus, methylation of me^0^ transforming ssDNA by DpnA should be crucial for exchange of plasticity islands. Conversely, the absence of DpnA should leave the resulting transformants sensitive to *Dpn*II (Fig. 1C), which should restrict the chromosome, killing the cell.

Recently, evidence was obtained that the inefficiency of plasmid transformation is presumably intrinsic to the transformasome, due to the competence-induced ssDNA-binding protein, SsbB. We showed that SsbB protects internalized ssDNA and creates a reservoir favoring chromosomal transformation [17]. In contrast, SsbB was found to antagonize plasmid transformation [17], strongly suggesting that the pneumococcal transformasome has evolved to optimize chromosomal but not plasmid transformation. This conclusion prompted us to explore and validate the above-described hypothesis that DpnA is crucial for exchange of plasticity islands [3].

In this study, we show that in the absence of DpnA, acquisition of large me^0^ heterologous regions is drastically reduced. We also demonstrate that σ^X^-dependent induction of *dpnA* during competence is required for full protection of me^0^ foreign DNA. We conclude that the competence-induction of *dpnA* and the methylation of me^0^ ssDNA by DpnA are crucial for protection of foreign me^0^ DNA, allowing exchange of pathogenicicy islands such as the capsule locus. We conclude that the recruitment of *dpnA* and *dpnB* (encoding *Dpn*II) into the competence regulon provides the pneumococcus with protection from me^0^ bacteriophage through the action of *Dpn*II, whilst maintaining the potential of acquisition of me^0^ pathogenicity islands (e.g., of DpnI origin) via the protective role of ssDNA methylation by DpnA.

## Results

### DpnA is critical for the efficient acquisition of me^0^ pathogenicity islands by DpnII strains

**Figure 3:**
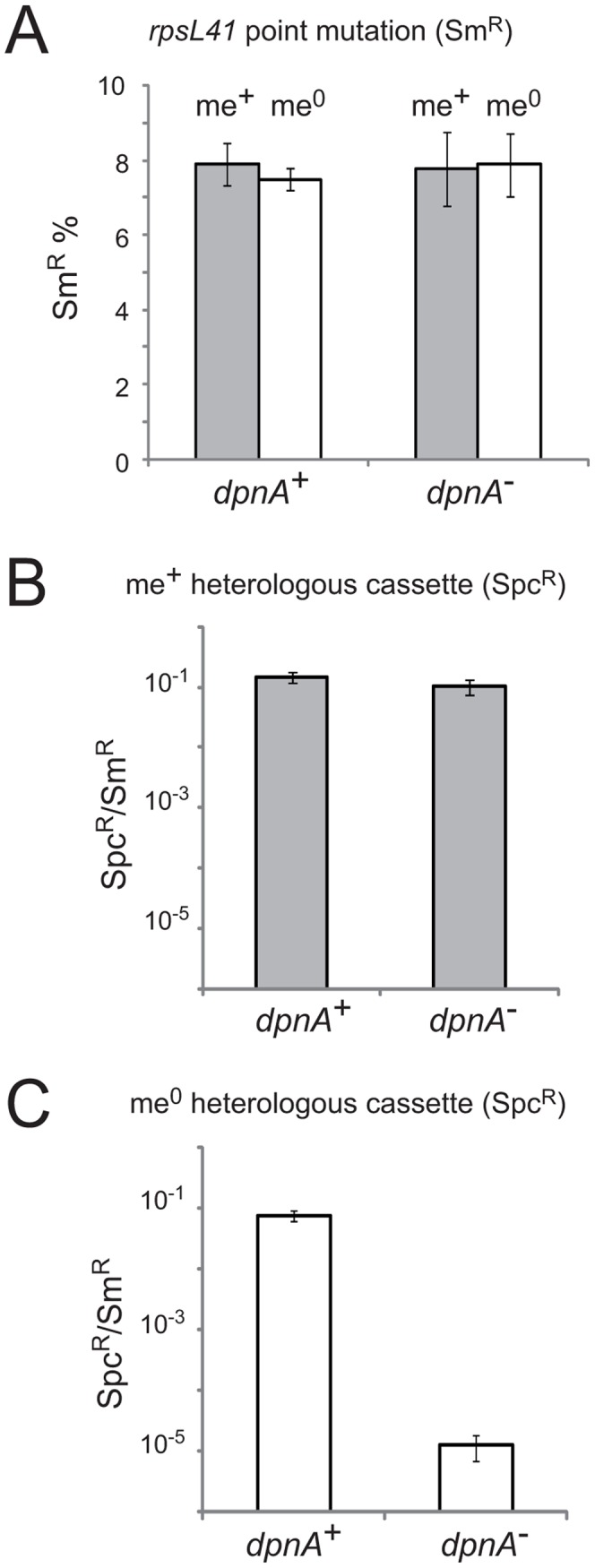
Methylation of ssDNA by DpnA is crucial for protection of heterologous me^0^ DNA from *Dpn*II restriction. (A) Transformation efficiency of point mutation *rpsL41* in recipients with and without DpnA. Dark grey and white columns represent respectively me^+^ and me^0^ donor DNA. Error bars are calculated from triplicate repeats of experiments (N = 3). Strains used: donor, TD153 (DpnI, *rpsL41*) and R3026 (DpnII, *rpsL41*); recipient, R3087 (DpnII) and R3088 (DpnII, *dpnA^−^*). (B) Transformation efficiency of me^+^ heterologous *cps2E*::*spc7* ^C^ cassette in *dpnA^+^* and *dpnA^−^* recipients. Values shown are normalized to an *rpsL41* point mutation also present on the donor DNA. Error bars are calculated from triplicate repeats of experiments (N = 3). Strains used: donor, R3026 (DpnII, *cps2E::spc7* ^C^, *rpsL41*); recipient, R3087 (DpnII) and R3088 (DpnII, *dpnA^−^*). (C) Transformation efficiency of me^0^ heterologous cassette in *dpnA^+^* and *dpnA^−^* recipients. See panel B legend. Strain used as donor, TD153 (DpnI, *cps2E::spc7* ^C^, *rpsL41*); same recipients as in panel B. See Fig. 1 for interpretations.

In order to validate our hypothesis that DpnA should be important for transfer of me^0^ heterologous pathogenicicy islands in DpnII strains, we created a series of isogenic strains possessing the full *dpnII* locus, or the *dpnII* locus with a previously characterized internal deletion of *dpnA* [13] (Fig. 2). We compared the transformation efficiency of cassettes on donor chromosomal DNA from either DpnI (me^0^) or DpnII (me^+^) origin in *dpnA* ^+^ and *dpnA* ^−^ competent cells. We began by confirming a previous conclusion, based on indirect comparisons between non-isogenic pairs of donor and recipient cells, that DpnA plays no role in transformation of a chromosomal point mutation [13] using *rpsL41* (conferring streptomycin resistance, Sm^R^) [18] (Fig. 3A).

We then tested the efficiency of transformation of a *cps2E*::*spc* cassette from either DpnI (me^0^) or DpnII (me^+^) donor DNA into DpnII recipients with or without *dpnA*. This transformation required integration of ∼8.7 kb of heterology within the capsule locus. Efficiency was compared to that obtained with the *rpsL41* Sm^R^ point mutation on the same donor DNA. As expected, there was no deficit in transformation efficiency in a *dpnA^−^* recipient when the donor DNA was me^+^ (Fig. 3B). In contrast, a >4-log deficit in transformation efficiency was observed when the donor DNA was me^0^ (Fig. 3C). This is because in presence of DpnA, prior methylation of ssDNA protects resulting transformants (as in Fig. 1B), and in absence of DpnA, the incorporated DNA remains me^0^, and resulting transformants are sensitive to *Dpn*II restriction (as in Fig. 1C). This result confirms that protection of heterologous me^0^ donor DNA from restriction by DpnA-mediated methylation is crucial for successful transfer.

### The number of GATC sites present in me^0^ heterologous DNA determines importance of DpnA-mediated protection

**Figure 4:**
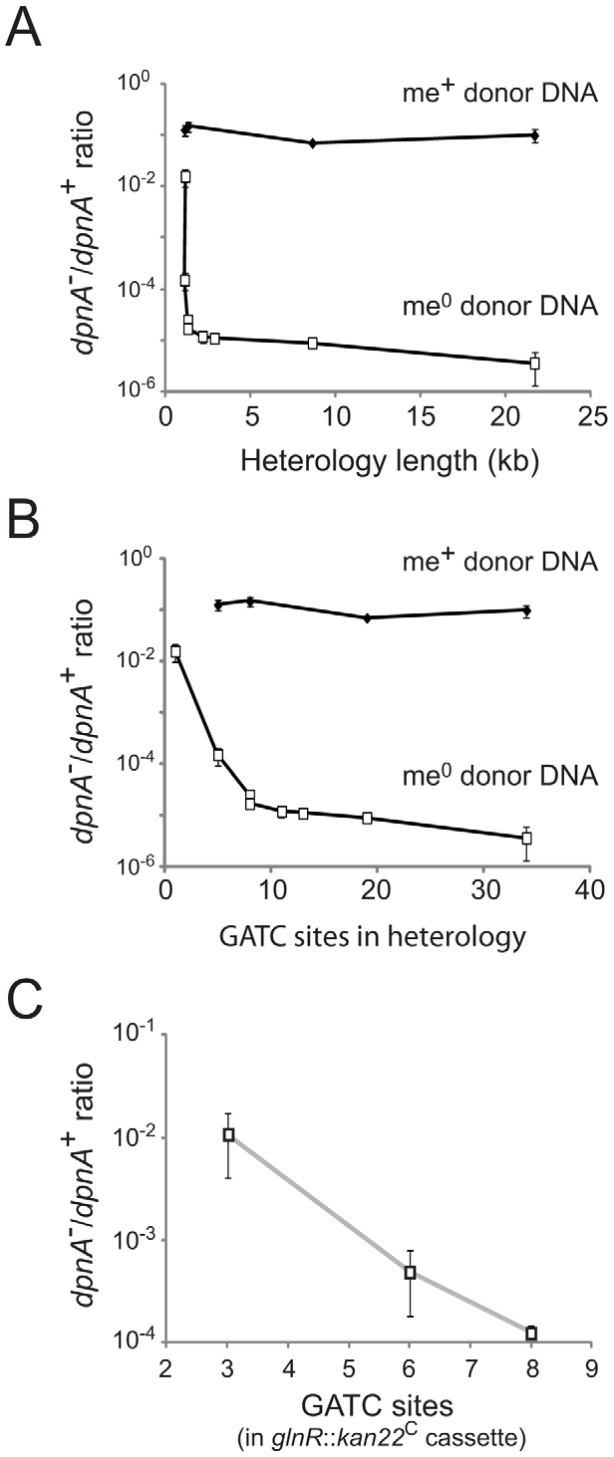
Importance of DpnA-mediated protection from restriction is dependent on the number of GATC sites present in the heterologous cassette. (A) Relationship between heterology length and *dpnA^−^* to *dpnA* ^+^ ratio. Ratios represent transformation efficiency in *dpnA^−^* compared to *dpnA^+^*. me^+^ donor DNA, closed diamonds. me^0^ DNA, open squares. Error bars are calculated from triplicate repeats of experiments (N = 3). Cassettes transferred and donor strains can be found in Table 1. Recipient strains: R3087 (DpnII); R3088 (DpnII, *dpnA^−^*); R3163 (DpnII) and R3164 (DpnII, *dpnA^−^*) were used for transfer of *fcsR*::*ermAM1* ^C^ to overcome antibiotic incompatibilities. To create the 21.7 kb heterology, the following recipient strains were used, which lacked the fully capsule locus: R3148 (DpnII, *cps*::*kan*) and R3149 (DpnII, *dpnA^−^*, *cps*::*kan*). (B) Relationship between number of GATC sites in donor heterologous cassette and *dpnA^−^* to *dpnA^+^* ratio. Same experiment as in panel A, but with ratios plotted against GATC sites in the heterology. (C) Comparison of transformation efficiency of *glnR*::*kan22* ^C^ cassettes with varying number of GATC sites. me^+^ donor DNA was not tested. Error bars as in panel A legend. Strains used: donor, R3154 (DpnI, *glnR*::*kan22* ^C (8)^, *rpsL41*), R3238 (DpnI, *glnR*::*kan22* ^C (3)^, *rpsL41*) and R3239 (DpnI, *glnR*::*kan22* ^C (6)^, *rpsL41*); recipient, R3087 (DpnII) and R3088 (DpnII, *dpnA^−^*).

The *cps2E*::*spc* cassette represents a large heterology, with 19 GATC sites available for *Dpn*II restriction. To determine whether DpnA was required to protect shorter regions of heterology, with fewer GATC sites, transfer of five further cassettes (Table 1) was compared. The results, presented as ratio of transformation frequency in *dpnA^−^* cells compared to *dpnA* ^+^ cells (after normalization to that of the Sm^R^ point mutation present on the same donor DNA), termed *dpnA* ^−/+^ ratio, show that longer heterologies are correspondingly more dependent on DpnA for protection (Fig. 4A). Indeed, most of the transfers result in a deficit in *dpnA^−/+^* ratio of 3 to 4 logs between me^+^ and me^0^ donor DNA, reinforcing the importance of DpnA for protection of these me^0^ heterologous cassettes.

**Table 1:** Information on heterology cassettes used in this study.

Cassette	Heterology length (bp)	GATC sites	Resistance	me^0^ donor (DpnI)†	me^+^ donor (DpnII)†
*fcsR*::*ermAM1* ^C^ *	1182	1	Ery^R^	R3147	n/a
*ciaR*::*spc119* ^A^	1146	5	Spc^R^	R1173	R3122
*endA*::*kan6* ^C^	1336	8	Kan^R^	R1173	R3122
CEP_x_ *-recA*	2204	11	Kan^R^	R3126	n/a
CEP_F_ *-licD1-licD2*	2900	13	Kan^R^	P503	n/a
*cps2E*::*spc7* ^C^ ‡	8651	19	Spc^R^	TD153	R3026
*cps2E*::*spc7* ^C^ §	21709	34	Spc^R^	TD153	R3026

* ^C^ and ^A^ indicate, respectively, the co-transcribed and reverse orientation of an inserted mini-transposon antibiotic resistance cassette with respect to the target gene.

† See Table S2.

‡ *cps2E*::*spc7* ^C^ cassette transformed into R6-derived strains R3087 and R3088 (with 8 kb deletion in capsule locus).

§ *cps2E*::*spc7* ^C^ cassette transformed into *cps*::*kan* mutant strains R3148 and R3149 (with 21 kb capsule locus deleted).

This relationship could simply reflect the increased probability of finding GATC sites with increasing heterologous segment length (Fig. 4B). To establish whether the number of GATC sites is important in determining the dependence on methylation of ssDNA by DpnA, we modified the *glnR*::*kan22* ^C^ cassette to create derivatives with the same heterology length (1,336 bp) but with 3 (*glnR*::*kan22* ^C (3)^), 6 (*glnR*::*kan22* ^C (6)^) or 8 (*glnR*::*kan22* ^C (8)^) me^0^ GATC sites. If the number of GATC sites present in the heterologous DNA determined the reliance of successful transfer on DpnA-mediated methylation of ssDNA, we expected that *glnR::kan22* ^C (3)^ should transfer into a *dpnA^−^* strain with higher efficiency than *glnR::kan22* ^C (6)^, which should in turn transfer with greater efficiency than the wild-type *glnR::kan22* ^C (8)^ cassette. The results confirm this prediction, showing that the *dpnA^−/+^* ratio of transformation frequencies is inversely proportional to the number of GATC sites in the heterology, with transformation efficiency in *dpnA^−^* recipient cells decreasing as GATC sites in donor heterology region increase (Fig. 4C). The number of GATC sites present in the heterologous donor DNA thus determines the importance of DpnA, suggesting that the ss-methylase is particularly important for the transfer of long heterologous regions of me^0^ DNA. We propose that absence of DpnA results in direct competition between DpnM and *Dpn*II for access to integrated me^0^ dsDNA following replication, where DpnM must methylate all GATC sites to protect the chromosome before *Dpn*II restricts a single one. As a result, increasing the number of heterologous GATC sites favors *Dpn*II in this competition, resulting in greater loss of transformant cells, since a greater proportion are destroyed by *Dpn*II.

### The vast majority of DpnA is produced during competence for genetic transformation

**Figure 5:**
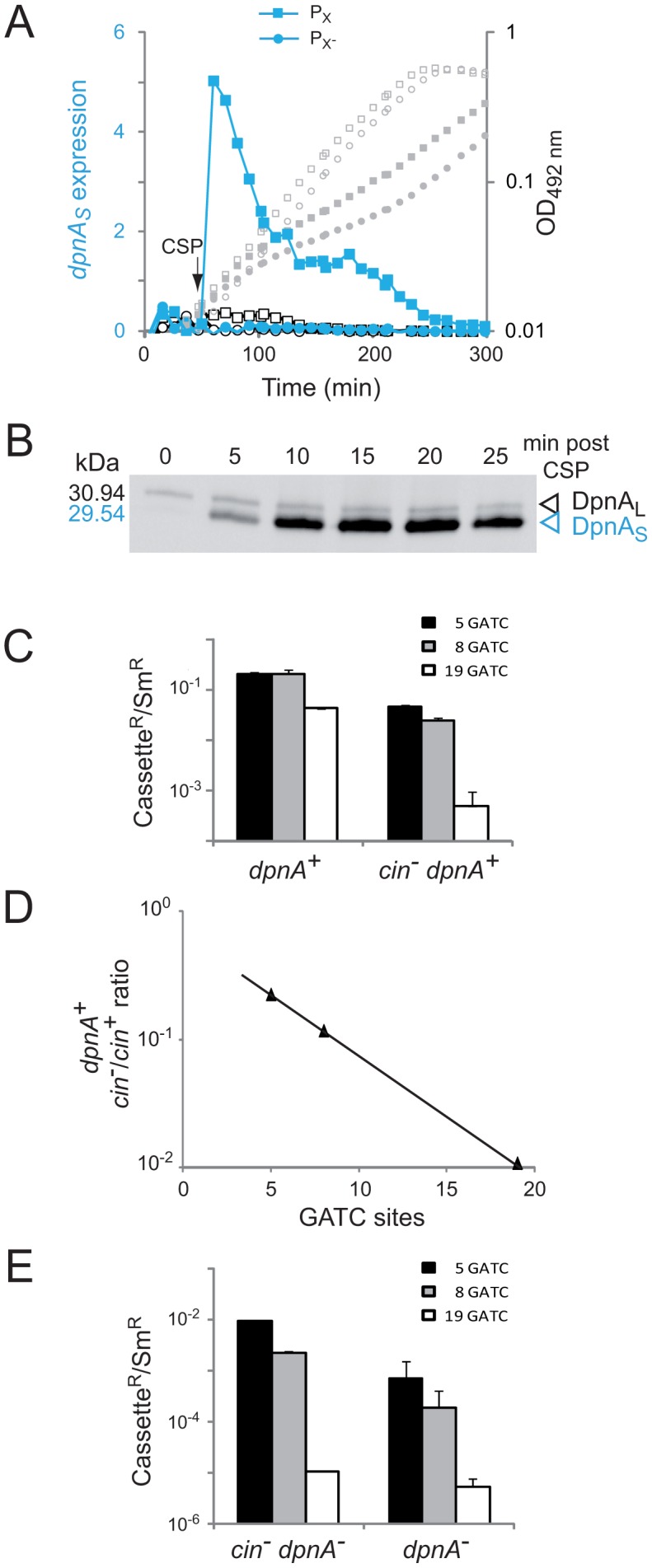
Induction of *dpnA* during competence is critical to ensure full protection of transforming heterologous DNA from restriction by *Dpn*II. (A) Expression of *dpnA_S_* is dependent on the *cin* box in P_X_. Expression (RLU/OD values) is represented by blue (with CSP) and black lines with symbols, whilst OD values are represented by grey symbols. The (P_X_)*-luc* reporter is represented by squares, and the mutated (P_X_-)-*luc* by circles. Closed shapes represent cultures which received CSP after 50 min incubation (arrow), whilst open shapes correspond to those without CSP. Strains used: R2323 ((P_X_)-*luc*) and R3233 ((P_X_-)-*luc*). (B) Expression of DpnA during competence. Whole extracts of cells containing a DpnA-SPA fusion were analyzed by Western-blot using anti-Flag antibodies (Text S1). Note that we routinely use these antibodies to detect many other pneumococcal proteins tagged with the SPA tag and do not observe the band we identify as DpnA_L_. Strain used: R3562 (DpnII, *dpnA-*SPA). (C) Transformation efficiencies of heterologous cassettes in *dpnA^+^* and *cin^−^ dpnA^+^* strains. *ciaR*::*spc119* ^A^ (5 GATC) in black, *endA*::*kan6* ^C^ (8 GATC) in grey, *cps2E*::*spc7* ^C^ (19 GATC) in white. Error bars are calculated from triplicate repeats of experiments (N = 3). Strains used: donor, TD153 (DpnI, *cps2E::spc7* ^C^, *rpsL41*) and R1173 (DpnI, *ciaR*::*spc119* ^A^, *endA*::*kan6* ^C^, *rpsL41*); recipient, R3087 (DpnII) and R3190 (DpnII, *cin^−^ dpnA^+^*). (D) Ratios of transfer efficiency of heterologous cassettes between *cin* ^−^ *dpnA^+^* and *cin* ^+^ *dpnA^+^* strains. (E) Transformation efficiencies of heterologous cassettes in *cin^−^ dpnA^−^* and *cin^+^ dpnA^−^* strains. Cassettes and error bars as in panel C. Strains used, donor as in panel C; recipient, R3088 (DpnII, *dpnA^−^*) and R3642 (DpnII, *cin^−^ dpnA^−^*).

Although it was previously shown that DpnA is specifically induced during competence for genetic transformation [11], the specific induction profile of DpnA was not established. Prior to determining the importance of DpnA competence induction for protection of me^0^ heterologous transforming DNA, we further explored the expression of DpnA during competence induction. Firstly, ectopic luciferase reporter fusion assays confirmed that the wild type ComX-dependent promoter, P_X_, was induced by competence-stimulating peptide (CSP) [19], whilst a promoter with a mutated *cin* box, P_X_-, was completely inactive (Fig. 5A). Secondly, a time course Western-blot showed that DpnA was expressed in two forms, a longer form, weakly constitutively expressed from P*_dpn_* (DpnA_L_), and a shorter form specifically expressed from P_X_ during competence (DpnA_S_) (Fig. 5B). The observation of both DpnA_L_ and DpnA_S_ proteins in competent pneumococcal cells establishes that the two forms previously observed in *E. coli* extracts [13] are biologically relevant. These results demonstrate that although a small proportion of DpnA is constitutively produced, the vast majority of DpnA is produced during competence for genetic transformation.

### Competence induction of DpnA is crucial for full protection of me^0^ heterologous DNA

The importance of DpnA competence induction for pathogenicicy island exchange was investigated by mutating P_X_ in the *dpnII* locus, and comparing the transfer efficiency of me^0^ cassettes in this recipient (*cin^−^ dpnA^+^*) to that in a *dpnA* ^+^ recipient. Results showed that induction of DpnA during competence was crucial for full protection and transfer of me^0^ heterologous DNA, as transformation efficiency of me^0^ cassettes decreased substantially in the *cin^−^ dpnA* ^+^ recipient strain (Fig. 5C). Furthermore, comparing the ratio of transfer between *cin* ^+^ *dpnA^+^* and *cin^−^ dpnA* ^+^ recipient strains showed that an increase in the number of heterologous GATC sites reinforced the importance of competence induction of DpnA (Fig. 5D). As noted above, this is likely due to the fact that DpnM and *Dpn*II compete directly for access to me^0^ GATC sites in the chromosome after integration of me^0^ heterologous DNA and replication.

It is of note that mutating the *cin* box in an otherwise *dpnA^−^* recipient (i.e., rendering the cell *cin^−^ dpnA^−^*) increased transformation efficiency compared to *cin^+^ dpnA^−^* cells (Fig. 5E). We attribute this increase to the non-induction of *dpnB* at competence, which results in lower levels of *Dpn*II, shifting the competition between DpnM and *Dpn*II for access to GATC sites in favor of the former, and thus increasing the probability that me^0^ dsDNA GATC sites will be protected after replication.

## Discussion

### A critical protective role of the ss-methylase DpnA for exchange of pathogenicicy islands

The primary role of the DpnII R-M system is to protect the pneumococcus from attack by me^0^ bacteriophage [13]. Here we show that the ss-methylase DpnA permits acquisition of me^0^ pathogenicicy islands (e.g., from DpnI pneumococci) via methylation of internalized foreign heterologous me^0^ ssDNA (Fig. 3C). Transformation of a 21.7 kb heterologous region is thus reduced by more than 4 logs in *dpnA* mutant cells. DpnA-dependent methylation occurring before as well as possibly after integration (i.e., at the heteroduplex stage; Fig. 1C) renders the incorporated dsDNA resistant to *Dpn*II after replication (Fig. 1B), and ensures the survival of the transformed cell, allowing exchange of pathogenicicy islands between DpnI and DpnII populations.

### Competence induction of DpnA is crucial for full protection of me^0^ heterologous DNA

A time course Western-blot following CSP addition revealed that the vast majority of DpnA is produced during competence for genetic transformation (Fig. 5B). The importance of competence induction of *dpnA* for acquisition of heterologous DNA was evaluated through inactivation of the *cin* box, bound by σ^X^, revealing a ∼100-fold reduction in transformation for a segment harboring 19 GATC sites (Fig. 5C and 5D). This reduction establishes that for such a large heterology ∼99% of the protection relied on DpnA molecules synthesized at competence.

It is of note that when comparing heterologous transfers in *dpnA* ^+^ and *cin* ^−^ *dpnA* ^+^ recipients to assess the impact of *dpnA* competence induction, we likely underestimate the true importance of this induction. We observed that the transformation efficiency in a *cin^−^ dpnA* ^+^ strain remains higher than in a *cin^+^ dpnA* ^−^ equivalent (compare Fig. 5C and 5E), and attribute this to two factors. Firstly, DpnA_L_ remains (weakly) constitutively expressed in *cin^−^ dpnA^+^* strains. Secondly, we have shown that *dpnB* (encoding *Dpn*II), as well as *dpnA*, is part of the competence regulon (Fig. 5E). As a result, mutating P_X_ reduces not only DpnA but also *Dpn*II levels, which should favor DpnM in the race against *Dpn*II, allowing survival of more recombinants and resulting in underestimation of the effect of non-induction of DpnA during competence.

Since the vast majority of DpnA is produced during competence (Fig. 5B), we conclude that the induction of DpnA is critical for full protection of heterologous ssDNA internalized during competence. We have shown DpnA-mediated protection to be especially important in the case of large pathogenicicy islands (Fig. 4A). The number of GATC sites, which are targeted by *Dpn*II, being proportional to length of heterologous regions and the abundance of these sites dictating the probability of restriction by *Dpn*II (Fig. 4) readily explain this observation.

### Evolutionary significance of the recruitment of *dpnA* and *dpnB* into the *com* regulon

The position of the P_X_ promoter downstream of *dpnM* (Fig. 2) results in induction of both *dpnA* and *dpnB*, but not *dpnM*, during competence. What could the biological relevance of this organization be? A first advantage we see in the recruitment of *dpnA* to protect transforming DNA is that the window of opportunity for DpnA methylation of heterologous ssDNA is long, existing between internalization of exogenous ssDNA, and the passage of the replication fork over the recombinant chromosome. In contrast, DpnM can only methylate heterologous DNA in the form of dsDNA incorporated into the chromosome, a much smaller window of opportunity. Our findings confirm this interpretation by showing that DpnM-mediated protection is inefficient, since in the absence of DpnA, the vast majority of transformants are lost due to *Dpn*II restriction (Fig. 3C). Moreover, competence induction of *dpnM* (i.e., to favor protection versus restriction) would be counter-productive for the cell, potentially increasing protection of me^0^ dsDNA phage and thus antagonizing the main role of *Dpn*II. DpnA affords an elegant solution to this problem, as it provides protection to transformation intermediates via ss-methylation, but cannot antagonize *Dpn*II-mediated phage destruction. Protecting long heterologous regions is of particular importance for the transfer of pathogenicity islands, such as the capsule locus which is generally considered the most important virulence factor of the pneumococcus. Capsule switching has been shown to be a prominent means of vaccine escape [20]. DpnA is likely to be crucial for DpnII cells to acquire serotypes from DpnI origin by transformation and, more generally, to allow evolution of the bacteria by acquisition and integration of foreign DNA.

As concerns the specific co-induction of *dpnB* with *dpnA* during competence, if *Dpn*II concentration increase in competent cells is similar to that observed for DpnA, then clearly it pushes the *Dpn*II/DpnM ratio toward restriction thus increasing the protection against dsDNA me^0^ bacteriophage, whilst maintaining the plasticity potential of the cell via DpnA.

### DpnI/DpnII R-M systems are not a barrier to genetic exchanges amongst pneumococci

The DpnI and DpnII R-M systems appear equally distributed in the pneumococcal population (Table 2). In the case of DpnI strains, no genetic barrier exists, as even when integrating me^+^ heterologous DNA (i.e., from DpnII), me^+^ dsDNA GATC sites are never produced in the host chromosome, which is never sensitive to *Dpn*I restriction. In the case of DpnII strains, we have shown that DpnA protects heterologous me^0^ ssDNA, allowing acquisition of foreign pathogenicicy islands and preventing formation of a genetic barrier between the two populations. We would therefore predict that DpnI/DpnII R-M systems are not a barrier to genetic exchanges amongst pneumococci, i.e., that other variable loci such as capsular serotype [5], capsule regulatory module [21] or CSP pherotype [22] would be randomly distributed in the DpnI and DpnII populations. This prediction proved to be correct as evidenced by the 19A and 19F capsule serotypes in both Dpn types (Table 2). As a Dpn switch should be lethal, since expression of *Dpn*I in a DpnII recipient would destroy the chromosome, and vice versa, we infer that the observed switches occurred in the other variable loci. The DpnI/DpnII split thus protects the pneumococcal species from both me^+^ and me^0^ bacteriophage, ensuring the survival of the pneumococcal species in the face of any bacteriophage attack, without creating an evolutionary barrier between the two sub-populations, thanks to the existence of and competence-induced production of DpnA.

**Table 2:** Presence of 19A and 19F serotypes in both DpnI and DpnII populations.

Isolate* (pneumococcal unless stated)	R-M system	CSP type	Capsule regulatory module†	Capsular type	Reference
D39	DpnI	1	blue (100; 75)	2	[37]
TIGR4	DpnI	2	red (75; 100)	4	[38]
Canada MDR 19A	DpnI	1	blue (89; 74)	19A	Unpublished
CDC3059-06	DpnI	1	blue (89; 74)	19A	[39]
TCH8431/19A	DpnI	1	blue (89; 73)	19A	[40]
Canada MDR 19F	DpnI	1	red (75; 97)	19F	Unpublished
Taiwan 19F-14	DpnI	1	red (75; 97)	19F	[39]
SP19-BS75	DpnI	1	red (75; 97)	19F	[41]
ST556	DpnI	1	red (75; 97)	19F	Unpublished
Hungary 19A-6	DpnII	1	blue (89; 73)	19A	[39]
G54	DpnII	1	red (75; 97)	19F	[41]
*S. mitis* SK321‡	DpnI	SK321§	n/a	n/a	Unpublished
*S. mitis* B6‡	DpnII	B6	n/a	n/a	[22]

* pneumococcal isolates represent assembled pneumococcal genomes. Dpn identity of other available sequenced pneumococcal genomes with known serotypes can be found in Table S1.

† capsule regulatory module identity determined by BLAST against *wze* sequence from blue and red references defined by Varvio and colleagues [21]. Values in brackets refer to % nucleotide sequence identity with D39 *wze* (blue module); followed by % identity with TIGR4 *wze* (red module).

‡ Two *S. mitis* sequenced isolates arbitrarily chosen to demonstrate presence of both *Dpn*I and *Dpn*II systems in *S. mitis* population.

§ 87% identity to CSP2.

### ss-methylases in other species

Although methylases specific for ssDNA such as DpnA appear to be rare, a few others exist. Both DpnI and DpnII are present in *Streptococcus mitis* (our observations), a species closely-related to *S. pneumoniae*, and we propose that DpnB/DpnA play a similar dual role of protection from bacteriophage attack and maintenance of genetic exchange. Supporting this notion, the *cin* box in the P_X_ *dpnA* promoter is perfectly conserved in a *S. mitis* DpnII strain [23] (our observations), suggesting that *dpnA* and *dpnB* form part of the competence regulon in this species as well. In *Bacillus centrosporus*, an analogous R-M system, BcnI, also possessing an ss-methylase, was identified [24]. If *B. centrosporus* were transformable, it would be interesting to investigate whether this ss-methylase plays a similar role to that of DpnA with respect to pathogenicicy/plasticity island exchange.

### Impact of R-M systems on transformation in other species

Competence for genetic transformation is widespread in bacteria [2], although few studies have addressed the effect of R-M systems on genetic plasticity in these species. In the naturally transformable species *Helicobacter pylori*, R-M systems antagonize transformation, restricting genetic plasticity [25]. However, due to the internalization of exogenous DNA in ss form, the authors remained unclear as to the mechanism involved in this antagonization, and stated in a recent review ‘Because restriction enzymes prefer dsDNA, they likely act prior to unwinding and perhaps extracellularly’ [26]. Our model of *Dpn*II-mediated restriction of transformant chromosomes formed after integration of me^0^ heterologous DNA (Fig. 1C) addresses this problem, highlighting the availability of fully sensitive me^0^ dsDNA in the transformant chromosome after replication, explaining how classical R-M systems can antagonize heterologous transformation. In contrast, we show that through programmed protection of foreign, heterologous me^0^ ssDNA by DpnA, the pneumococcal DpnII R-M system actively promotes genetic plasticity by favoring acquisition of heterologous me^0^ cassettes. This, along with the lack of identified ss-methylases in other transformable species, suggests that not all competent species have developed R-M systems that protect from bacteriophage attack, whilst at the same time maintaining the potential for genetic plasticity, and thus adaptation to environmental stresses by acquisition of virulence loci. It is likely that the need for such genetic flexibility is species-specific, since the pneumococcus, a pathogen and common commensal of humans constantly exposed to host, antibiotic and vaccine stresses, may need to maintain its propensity for rapid adaptation more so than another species occupying a different niche. The relative loss of genetic plasticity afforded by classic R-M systems may not be problematic for bacterial species that are not readily exposed to such selective pressures, and as such do not need to maintain a high level of genetic plasticity.

### Alternative defense systems and maintenance of genetic exchanges

Other systems of defense against attack by foreign DNA exist, such as the clustered, regularly interspaced short palindromic repeat loci (CRISPR), which are sequence-specific defense mechanisms against bacteriophage, and have been shown to constitute a barrier to genetic diversity by horizontal gene transfer [27]. The pneumococcus does not possess CRISPR sequences, but a recent study artificially inserted CRISPR loci into the pneumococcal genome, and demonstrated that CRISPR interference blocked genetic plasticity by transformation in an *in vivo* model [28]. The authors concluded that CRISPR interference can limit the adaptability of a pathogen to a particular stress by limiting genetic plasticity. In contrast to our findings on the DpnII R-M system, no known mechanism exists to negate the antagonistic effect of CRISPR on genetic plasticity. Since we suggest that the pneumococcus appears to preferentially develop mechanisms that promote genetic diversity [3], the absence of CRISPR loci in this pathogen is not surprising. Indeed, despite the negative effect of CRISPR on genetic plasticity, the pneumococcus demonstrated the genetic flexibility required to spontaneously eject the CRISPR sequences when under environmental stress in the *in vivo* model, thus permitting a number of clones to adapt and survive [28]. It is unclear whether this ejection would be possible in bacteria that naturally possess CRISPR sequences, and do not favor adaptation by genetic plasticity to the same extent as the pneumococcus.

Bacterial pathogens must overcome two types of threat to survive and thrive, attack by foreign DNA (in the form of bacteriophage), and defenses mounted by host factors, antibiotics and vaccines. The pneumococcal DpnII system is an elegant system achieving both these ends. The *Dpn*II restrictase protects the cell from me^0^ dsDNA bacteriophage attack, whilst the DpnA ss-methylase maintains the pneumococcus' extraordinary potential for genetic plasticity, allowing adaptation to and evasion of defenses, natural or artificial, mounted by the human host upon pneumococcal infection.

### Concluding remarks

The question of why bacteria internalize exogenous DNA has been discussed at length [29]–[32]. The two credible suggestions are the use of DNA for genome maintenance via template-directed repair, or to promote genetic diversity via integration of exogenous DNA into the host genome. Here we show that competence induction of a locus coupling an ss-methylase to a restrictase is crucial for protection of heterologous exogenous DNA in the pneumococcus. We do not see how this protection could participate in genome maintenance, whereas it can clearly facilitate exchange of pathogenicity/plasticity islands. As a result, we consider this finding as the first direct evidence that *S. pneumoniae* can take up foreign DNA with the specific goal of integrating it into its chromosome via recombination, to promote genetic diversity.

## Materials and Methods

### Bacterial strains, plasmids, growth and transformation conditions

*S. pneumoniae* strain growth and transformation were carried out as described [33]. Strain, plasmid and primer information can be found in Table S2. Recipient strains were rendered *hex^−^* by insertion of the *hexA*::*ermAM* cassette as described [34], negating any effect of the mismatch repair system on transformation efficiencies [35]. Antibiotics were used at the following concentrations; Chloramphenicol 4.5 µg mL^−1^, Erythromycin 2 µg mL^−1^, Kanamycin 250 µg mL^−1^, Spectinomycin 200 µg mL^−1^, Streptomycin 200 µg mL^−1^.

### Replacement of *dpnI* locus by *dpnII* (with or without *dpnA*) in R800 background

The *dpnI* locus was replaced with the *dpnII* locus in laboratory strain R800 by Janus [36]. Briefly, the *dpnI* locus from R800 was amplified by PCR with primers dpnCup and dpnDdo, possessing *Bam*HI and *Pst*I restriction sites respectively, and cloned into pGBDU. The resulting plasmid (pGBDU-*dpn*I) was digested by *Cla*I and *Sac*I restriction enzymes, deleting a 180 bp internal fragment of *dpnC*, encoding the *Dpn*I restrictase. The Janus cassette was amplified with primers kan5c and 7 sac, possessing *Cla*I and *Sac*I sites, and ligated into pGBDU-*dpnC* ^−^, to give pGBDU-*dpn*I-Janus. The resulting plasmid was transformed into R981, with kanamycin selection, creating R2888. The Janus cassette was replaced with *dpnII* locus either wildtype (G54 donor DNA) or *dpnA^−^* (1135 donor DNA [13]) creating R2980 and R2981, respectively. The resulting strains were rendered Sm^S^ by transformation with a wild-type *rpsL* PCR fragment to remove the Sm^R^ point mutation *rpsL1*, creating R2992 and R2993, respectively.

### Calculation of *dpnA* ^−/+^ ratio

To calculate *dpnA^−^* ^/+^ ratios, the transformation efficiency of a specific cassette into *dpnA^−^* and *dpnA^+^* recipients was divided by the number of transformants by total number of colony forming units (cfu) obtained. This value was then normalized against a point mutation transformed on the same donor DNA (*rpsL41*, conferring Sm^R^), unaffected by absence of DpnA. The resulting transformation efficiency for a *dpnA^−^* recipient was divided by the same value for the same cassette in a *dpnA^+^* recipient to give the *dpnA^−/^* ^+^ ratio. A ratio of 1 indicates no effect of absence of DpnA on transformation efficiency, whilst a ratio of <1 indicates a loss of transformation efficiency in a *dpnA^−^* recipient strain.

### Miscellaneous methods

Further methods used in this study can be found in the Supporting Information. This file contains detailed descriptions of the cassettes used in this study, reporter fusions used to measure transcription of the P_X_ promoter, creation of the *cin^−^* mutated *dpnA* promoter and construction of *dpnA-*SPA.

## Supporting Information

Table S1 Dpn identity of sequenced pneumococcal genomes with known serotypes.
(DOCX)

Table S2 Strains, plasmids and primers used in this study.
(DOCX)

Text S1 Supporting Materials and Methods. Heterologous Cassettes, Reducing the Number of GATC Sites in *glnR*::*kan22* ^C^, Measuring Transcription from the P_X_ *dpnA* Promoter, Creation of *cin^−^* Mutated P_X_ Promoter at the *dpnII* Locus, Creation of DpnA-SPA, Western-blot analysis of DpnA expression.
(DOCX)
