# Supplementary material for: Programmed Protection of Foreign DNA from Restriction Allows Pathogenicity Island Exchange during Pneumococcal Transformation
Source: PLoS Pathog. 2013 Feb 14;9(2):e1003178. doi: 10.1371/journal.ppat.1003178 (PMC3573125; doi:10.1371/journal.ppat.1003178)
Supplement: Table S1 — Dpn identity of sequenced pneumococcal genomes with known serotypes. (DOCX) [file ppat.1003178.s001.docx]

| **Table S1.** Dpn identity of sequenced pneumococcal genomes with known serotypes. | | | | | | | |
| --- | --- | --- | --- | --- | --- | --- | --- |
|  | | | | |  |  |  |
|  | | | | |  |  |  |
| Strain/genome ref. | Dpn identity* | Serotype | Reference |  |  |  |  |
|  |  |  |  |  |  |  |  |
| A45 | DpnI | 3 | [14] |  |  |  |  |
| 07-2838 | DpnI | 3 | [14] |  |  |  |  |
| 02-1198 | DpnI | 3 | [14] |  |  |  |  |
| OXC141 | DpnI | 3 | [15] |  |  |  |  |
| SV36 | DpnI | 3 | [16] |  |  |  |  |
| CDC1873-00 | DpnI | 6A | [14] |  |  |  |  |
| SP6-BS73 | DpnI | 6A | [15] |  |  |  |  |
| SP9-BS68 | DpnI | 9 | [15] |  |  |  |  |
| SP195 | DpnI | 9V | [14] |  |  |  |  |
| England 14 | DpnI | 14 | Unpublished |  |  |  |  |
| INV200 | DpnI | 14 | [15] |  |  |  |  |
| CCRI 1974 | DpnI | 14 | [17] |  |  |  |  |
| SP14-BS292 | DpnI | 14 | [15] |  |  |  |  |
| SP14-BS69 | DpnI | 14 | [15] |  |  |  |  |
| CGSP14 | DpnI | 14 | [18] |  |  |  |  |
| JJA | DpnI | 14 | [14] |  |  |  |  |
| SP23-BS72 | DpnI | 23F | [15] |  |  |  |  |
| SV35 | DpnI | 23F | [16] |  |  |  |  |
|  |  |  |  |  |  |  |  |
| P1041 | DpnII | 1 | [14] |  |  |  |  |
| P1031 | DpnII | 1 | [14] |  |  |  |  |
| INV104B | DpnII | 1 | [15] |  |  |  |  |
| 06-1370 | DpnII | 1 | [14] |  |  |  |  |
| NCTC7465 | DpnII | 1 | [14] |  |  |  |  |
| 70585 | DpnII | 5 | [14] |  |  |  |  |
| 670-6B | DpnII | 6B | [15] |  |  |  |  |
| MLV-016 | DpnII | 11A | [14] |  |  |  |  |
| AP200 | DpnII | 11A | Unpublished |  |  |  |  |
| SP11-BS70 | DpnII | 11A | [15] |  |  |  |  |
| CDC0288-04 | DpnII | 12F | [14] |  |  |  |  |
| SP18-BS74 | DpnII | 18 | [15] |  |  |  |  |
|  | | | | |  |  |  |
|  | | | | |  |  |  |
| *Dpn identity determined by BLAST against pneumococcal genomes in NCBI database | | | | | | | |
