# Supplementary material for: Programmed Protection of Foreign DNA from Restriction Allows Pathogenicity Island Exchange during Pneumococcal Transformation
Source: PLoS Pathog. 2013 Feb 14;9(2):e1003178. doi: 10.1371/journal.ppat.1003178 (PMC3573125; doi:10.1371/journal.ppat.1003178)
Supplement: Table S2 — Strains, plasmids and primers used in this study. (DOCX) [file ppat.1003178.s002.docx]

|  | | | |
| --- | --- | --- | --- |
| \| **Table S2.** Strains, plasmids and primers used in this study. \| \|  \| \| --- \| --- \| --- \| \|  \|  \|  \| \| ***S. pneumoniae*  strain** \| **Genotype/Description** \| **Source Reference** \| \|  \|  \|  \| \| D39 \| Serotype 2 *cps* locus (21,709 bp) \| NCTC 7466 \| \| G54 \| *dpnII* \| [19] \| \| 1135 \| *dpnII*, *dpnA^-^* \| [20] \| \| TD153 \| D39 *rpsl41*, *cps2E*::*spc7*^C*^, *dpn*I; Sm^R^, Spc^R†^ \| This study \| \| P501 \| R6 derivative strain which grows independently of the essential nutrient choline \| [5] \| \| P503 \| P501 *rpsL41*, CEP_F_*-licD1-licD2*, *dpn*I; Sm^R^, Kan^R^ \| This study \| \| R800 \| Non-capsulated D39 derivative, with 8,651 bp deletion in the *cps* locus \| [10] \| \| R981 \| R800 *rpsL1*; Sm^R^ \| [21] \| \| R1173 \| R800 Δ*comC, rpsL1*, *ciaR*::*spc119*^A^, *endA*::*kan6*^C^, *dpn*I; Sm^R^, Spc^R^, Kan^R^ \| This study \| \| R2888 \| R800 *dpnC*::*Janus, rpsL1*; Kan^R^, Sm^S^ \| This study \| \| R2980 \| R800 *dpnII, rpsL1*; Sm^R^ \| This study \| \| R2981 \| R800 *dpnII, dpnA^-^, rpsL1*; Sm^R^ \| This study \| \| R2992 \| R800 *dpnII* \| This study \| \| R2993 \| R800 *dpnII, dpnA^-^* \| This study \| \| R3026 \| R800 *rpsL41****,*** *cps2E*::*spc7*^C^, *dpn*II; Sm^R^, Spc^R^ \| This study \| \| R3087 \| R800 *hexA*::*ermAM*, *dpn*II; Ery^R^ \| This study \| \| R3088 \| R800 *hexA*::*ermAM*, *dpn*II, *dpnA^-^*; Ery^R^ \| This study \| \| R3122 \| R800 *rpsL41*, *ciaR*::*spc119*^A^, *endA*::*kan6*^C^*, dpn*II; Sm^R^, Spc^R^, Kan^R^ \| This study \| \| R3126 \| R800 Δ*comC*, *recA-*SPA, CEP_x_*-recA*, *rpsL41*, *dpn*I; Kan^R^, Sm^R^ \| This study \| \| R3147 \| R800 Δ*comC*, *fcsR*::*ermAM1*^C^, *rpsL41*, *dpn*I; Ery^R^, Sm^R^ \| This study \| \| R3148 \| R800 *cps::kan,* *hexA*::*ermAM, dpn*II; KanR, EryR \| This study \| \| R3149 \| R800 *cps::kan,* *hexA*::*ermAM, dpn*II *dpnA^-^*; Kan^R^, Ery^R^ \| This study \| \| R3154 \| R800 *∆comC*, *glnR*::*kan22*^C (8)^, *rpsL41*; Kan^R^, Sm^R^ \| This study \| \| R3163 \| R800 *hexA*::*spc*, *dpn*II; Spc^R^ \| This study \| \| R3164 \| R800 *hexA*::*spc*, *dpn*II, *dpnA^-^*; Spc^R^ \| This study \| \| R3190 \| R800 *hexA*::*ermAM*, *dpn*II, *cin^-^ dpnA*^+^; Ery^R^ \| This study \| \| R3230 \| R800 Δ*comC*, *glnR*::*kan22*^C^, *rpsL41*, *dpn*I; Kan^R^, Sm^R^ \| This study \| \| R3232 \| R800 Δ*comC*, (P_X_)*-luc*, *dpn*I; Cm^R^ \| This study \| \| R3233 \| R800 Δ*comC*, (P_X_-)*-luc*, *dpn*I; Cm^R^ \| This study \| \| R3238 \| R800 Δ*comC*, *glnR*::*kan22*^C (3)^, *rpsL41*, *dpn*I; Kan^R^, Sm^R^ \| This study \| \| R3239 \| R800 Δ*comC*, *glnR*::*kan22*^C (6)^, *rpsL41*, *dpn*I; Kan^R^, Sm^R^ \| This study \| \| R3478 \| R800 *hexA*::*ermAM*, *dpn*II, *dpnA*-SPA; Ery^R^ \| This study \| \| R3562 \| R800 *hexA*::*ermAM*, *comA*::*kan, dpn*II, *dpnA*-SPA; Ery^R^, Kan^R^ \| This study \| \| R3642 \| R800 *hexA*::*ermAM*, *dpn*II, *cin*^-^ *dpnA^-^*; Ery^R^ \| This study \| \|  \|  \|  \| \| **Plasmids** \|  \|  \| \|  \|  \|  \| \| pEMcat \| Plasmid carrying Cm^R^ in a minitransposon cassette for *mariner* mutagenesis; Cm^R^ \| [9] \| \| pGBDU-*dpn*I \| pGBDU-derived plasmid with *dpn*I locus present \| This study \| \| pK3 \| pUC57-derived plasmid carrying Kan^R^ cassette and flanks based on pR410 sequence with 5 GATC sites silently mutated; Kan^R^, Ap^R^ \| Genscript, USA \| \| pK6 \| pUC57-derived plasmid carrying Kan^R^ cassette and flanks based on pR410 sequence with 2 GATC sites silently mutated; Kan^R^ Ap^R^ \| Genscript, USA \| \| pR424 \| Plasmid carrying an *ssbB*::*luc* fusion; Cm^R^ \| [11] \| \| pR475 \| pR424-derived plasmid with 500 bp homology to *dexB* present upstream of promoter insertion site \| This study \| \| pR475-T1T2 \| pR475-derived plasmid with T1T2 terminator between *dexB* and promoter insertion site \| This study \| \| pGBDU-*dpn*I-Janus \| pGBDU-derived plasmid with *dpn*I locus present, and with 180 bp fragment of *dpnC* replaced by Janus cassette \| This study \| \| pR475-T1T2-P_X_ \| pR424-derived plasmid with 500 bp homology to *dexB* present upstream of T1T2 terminator, P_X_  *dpnA* promoter driving *luc* gene expression; Cm^R^ \| This study \| \| pR475-T1T2-P_X_- \| pR424-derived plasmid with 500 bp homology to *dexB* present upstream of T1T2 terminator, P_X_- (i.e., *cin* box mutated) *dpnA* promoter driving *luc* gene expression; Cm^R^ \| This study \| \| pUC57-dpnA-SPA \| pUC57-derived plasmid containing *dpnA-SPA* construct for tagging *dpnA* without selection; Ap^R^ \| This study \| \| pUC57-P_X_*-* \| pUC57-derived plasmid for mutation of native *cin* box in P_X_ *dpnA* promoter; Ap^R^ \| Genscript, USA \| \| pR410 \| pEMcat derivative plasmid carrying a minitransposon Kan^R^ cassette for *mariner* mutagenesis; Kan^R^ \| [4] \| \|  \|  \|  \| \| **Primers** \| **Primer sequence^‡^** \|  \| \|  \|  \|  \| \|  \|  \|  \| \| CJ56 \| GCCGTCGACCATAAGCTCCTCCTAATTTA \| This study \| \| CJ57 \| GTGCCATGGTCGTCCTTGATTAACTTTAT \| This study \| \| CJ75 \| CTCAATCTTTTTAATTCTTTTGGG \| This study \| \| CJ76 \| TGAGTGACTATGCAGGTAACTGGC \| This study \| \| CJ130 \| GCGCTCGAGAATTTAATAGAGTGAGGTGAATT \| This study \| \| CJ131 \| GCGGGATCCTACTTATATTCGTTATTTTTCATAAT \| This study \| \| CJ139 \| GCGGGATCCTACTTAGTCGACTTATTTTTCATAATTTGTGACTATAA \| This study \| \| CJ159 \| GCGCCATGGATTGCAAGGAAGAGGTTGGAGGCAG \| This study \| \| CJ160 \| CGCAAGCTTTATCATCGCAACGGTGTCTATGGTT \| This study \| \| CJ162 \| GCGCTCGAGGTTTGTTTCATTTTCTGCCTCCAA \| This study \| \| CJ163 \| GCGCCATGGTCAGGTCACTACTTGTCATCGTCATCC \| This study \| \| CJ180 \| GCGGAATTCTAAGAATCAATTTAATGTTCCATA \| This study \| \| CJ181 \| GCGCTCGAGATGGAAAAGAGAAGATGGAAAAAG \| This study \| \| glnR1 \| ATGGACGCTATCGTGAAACAACC \| This study \| \| glnR2 \| GCGTTATTTCCTTCTGCATCAAAC \| This study \| \| cpsC1 \| AGAACAAAACACGATAGAAATCG \| This study \| \| cpsF1 \| GCCATCATAATCGCAATTTG \| This study \| \| dpn1 \| GCAGGAAAACTCGACGATGT \| This study \| \| dpn2 \| CTGCTTCCTAAGTTTGGTGCC \| This study \| \| dpnCup \| GCGGGATCCAACCTTTACCATGTTTGTCACA \| This study \| \| dpnDdo \| CGGCTGCAGTCTGCATCAAGAACAATTTCACA \| This study \| \| kan5c \| CCCATCGATGTTTGATTTTTAATGGATAATG \| This study \| \| 7sac \| TTTGAGCTCAGAGACCTGGGCCCCTTTCC \| This study \| \| licD1F \| GCGCCATGGAGTCTACTTGTCTTCACAAAAG \| This study \| \| licD2R \| GCGGGATCCGCCATACATCTGGGTTAAAA \| This study \| \|  \|  \|  \| \|  \|  \|  \| \| ^*C^ and ^A^ indicate, respectively, the co-transcribed and reverse orientation of an inserted mini-transposon \| \|  \| \| antibiotic resistance cassette with respect to the target gene \| \|  \| \| ^†R^, Resistance; Ap, ampicillin; Cm, chloramphenicol; Ery, erythromycin; Kan, Kanamycin; Sm, streptomycin; \| \|  \| \| Spc, spectinomycin \|  \|  \| \| ^‡^Underlined bases indicate restriction sites used for cloning in this study \| \|  \| |  |  |  |
