# Supplementary material for: Programmed Protection of Foreign DNA from Restriction Allows Pathogenicity Island Exchange during Pneumococcal Transformation
Source: PLoS Pathog. 2013 Feb 14;9(2):e1003178. doi: 10.1371/journal.ppat.1003178 (PMC3573125; doi:10.1371/journal.ppat.1003178)
Supplement: Text S1 — Supporting Materials and Methods. Heterologous Cassettes, Reducing the Number of GATC Sites in glnR::kan22 C, Measuring Transcription from the PX dpnA Promoter, Creation of cin− Mutated PX Promoter at the dpnII Locus, Creation of DpnA-SPA, Western-blot analysis of DpnA expression. (DOCX) [file ppat.1003178.s003.docx]

**Supporting Information**

Programmed Protection of Foreign DNA from Restriction Allows Pathogenicity Island Exchange during Pneumococcal Transformation

**Johnston et al., 2012**

**Text S1**

Heterologous Cassettes

*ciaR*::*spc119*^A^ and *endA*::*kan6*^C^ cassettes have previously been described [1,2], as has the CEP_X_-*recA* construct in strain R3126 [3]. *Mariner* mutagenesis was carried out as previously described [4]. Primers used to create *cps2E*::*spc7*^C^, *fcsR*::*ermAM1*^C^ and *glnR*::*kan22*^C^ *mariner* mutants were cps2C1-cps2F1, CJ75-CJ76, and glnR1-glnR2, respectively. *cps2E*::*spc7*^C^, *fcsR*::*ermAM1*^C^ and *glnR*::*kan22*^C^ *mariner* cassettes were inserted after bases 949, 372 and 55, with respect to the ATG of each gene.

The CEP_F_-*licD1-licD2* construct, present in the strain P503, based on P501 [5], was created as follows. Firstly, the P*_fcsK_* promoter [6] from R800 was amplified by PCR with primers CJ56-CJ57, possessing *Sal*I and *Nco*I sites. The *licD1-licD2* genes were amplified by PCR from R800 with primers licD1F- licD2R, possessing *Nco*I and *Bam*HI sites. These two fragments were digested and ligated into a pCEP plasmid [7] variant (possessing a T1T2 terminator upstream of the promoter insertion region, separated by a *Sal*I site) previously digested by *Sal*I and *Bam*HI, and transformed into P501. Deletion of the capsule locus in R3148 and R3149 was accomplished by transfer of a *cps*::*kan* cassette [8]. The details of antibiotic resistance cassettes used in this study can be found in Table 1. All heterology cassettes were transformed into strains R3087 (*dpnA^+^*) and R3088 (*dpnA^-^*) to compare transformation efficiencies, except *fcsR*::*ermAM1*^C^, which was transferred into strains R3163 (*dpnA^+^*) and R3164 (*dpnA^-^*), to overcome antibiotic incompatibilities.

Reducing the Number of GATC Sites in *glnR*::*kan22*^C^

The wild-type *glnR*::*kan22*^C^ cassette in strain R3154 has 8 GATC sites in the heterologous region. In order to create a *glnR*::*kan22*^C^ cassette with 3 or 6 GATC sites, two constructs were synthesized (Genscript) with GATC sites #1 (changed to CATC) and #8 (→CATC), or #1 (→CATC), #4 (→GACC), #5(→GACC), #6 (→CATC) and #8 (→CATC) of the heterologous region silently mutated, respectively. These mutated cassettes were present on the pUC57-derived plasmids pK3 and pK6. In order to swap these cassettes with the wild-type *glnR::kan22*^C^ cassette, R3154 was transformed with the pEMcat plasmid [9] to replace *glnR*::*kan22*^C^ with *glnR*::*cat22*^C^ in R3230. This strain was then transformed with the pK3 and pK6 plasmids to create R3238 and R3239, respectively.

Measuring Transcription from the P_X_ *dpnA* Promoter

In order to determine the transcription levels of P_X_, an ectopic expression platform was created by digestion of plasmid pR424 [10] with *Bam*HI and *Hind*III, and ligation with a 508 bp fragment of the *dexB* gene (amplified by primers dexB1-dexB2). The resulting plasmid (pR475) was digested by *Xho*I-*Bsg*I, and a T1T2 transcription terminator [10], amplified by primers T1T2bsg and T1T2xho was ligated into the vector downstream of the *dexB* homology, to block read-through from *dexB,* creating the plasmid pR475-T1T2. In this study, two promoter regions were amplified by PCR, digested by *Xho*I-*Bam*HI and ligated into the vector between T1T2 and *luc*. These were the wild-type P_X_ promoter and a promoter with a mutated *cin* box, P_X_- (see below), using primer pairs CJ130-CJ131 and CJ130-CJ139, respectively. These non-replicative vectors were then transformed into *S. pneumoniae*, resulting in insertion-duplication at the *dexB* gene, creating a platform to measure expression of promoter fragments. Transformants were selected by resistance to chloramphenicol (Cm^R^), creating strains R3232 and R3233 respectively. To determine transcription levels, luminometry experiments were carried out as previously described [11].

Creation of *cin^-^* Mutated P_X_ Promoter at the *dpnII* Locus

To mutate the *cin* box in P_X_ without disrupting the *dpnII* locus, a DNA fragment was synthesized (Genscript) with the mutated *cin* box at the centre and 500 bp of flanking DNA from the *dpnII* locus at either side on a pUC57 vector. The central 6 bases of the *cin* box (AACGAATA) were replaced by a *Sal*I restriction site (AAGTCGAC). This plasmid was transformed into R3087 and R3088 to mutate the *cin* box in both *dpnA*^+^ and *dpnA*^-^ contexts. Transformation was done without selection, and 20 transformant clones were assayed for successful mutation by PCR with primers dpn1-dpn2, followed by *Sal*I digestion, creating strains R3190 and R3642 respectively.

Creation of DpnA-SPA

A SPA tag was added, in frame, to 3’ end of *dpnA* orf to allow Western blotting via the FLAG tag [12]. A 500 bp region of *dpnA*, ending with the base prior to the stop codon, was amplified by PCR with primers CJ180 and CJ162 possessing *Eco*RI and *Xho*I sites. The SPA tag was amplified with primers CJ181 and CJ163, possessing *Xho*I and *Nco*I sites. A 500 bp region of *dpnA*-*dpnB* starting from the stop codon of *dpnA* was amplified with primers CJ159 and CJ160, possessing *Nco*I and *Hind*III sites. These PCR fragments were digested and co-ligated into a pUC57 plasmid digested *Eco*RI and *Hind*III, to create a plasmid containing a fragment of DNA with the SPA tag flanked on either side by 500 bp homology to the *dpnII* locus. This plasmid (pUC75-dpnA-SPA) was transformed into R3087 without selection, and clones were screened by PCR with dpn1 and dpn2 primers for insertion of the SPA tag, creating R3478. The *comA* gene was mutated by transfer of a previously-described *comA*::*kan* cassette [13], creating R3562.

Western-blot analysis of DpnA expression

For Western-blot analysis of DpnA expression, R3562 was grown in 50 ml C+Y medium at 37°C to 0D_550nm_ 0.1, and CSP (25 ng mL^-1^) added. The culture was then incubated at 37°C for a further 30 min, with 5 ml culture samples taken every 5 min (including immediately prior to CSP addition, t=0). Total cell content was collected by centrifugation, and pellets were resuspended in 300 µl 1x Tris (10 mM, pH 8.0) -EDTA (1 mM) buffer and lysed for 10 min at 37°C after addition of 8 µl DOC (0.25%)-SDS (0.5 %). 100 µl loading buffer were added and the suspension was incubated for 5 min at 85°C before loading onto a 4-12% acrylamide-SDS gel (Invitrogen), and allowed to migrate form 50 min at 200V. Proteins were transferred onto nitrocellulose membrane using an iBlot system (Invitrogen). Membranes were incubated overnight in 10% milk solution (in 1 x TBS + 0.1% Tween-20) at 4°C, before being incubated in 5% milk solution with 1/5,000 anti-FLAG monoclonal antibody (Sigma). Membranes were then washed four times in 1 x TBS + 0.1% Tween-20, and incubated in 5% milk solution with 1/15,000 anti-rabbit IgG monoclonal antibody (Sigma). Membranes were then washed four times in 1 x TBS + 0.1% Tween-20, and the ECL^TM^ Prime Western Blotting Detection System (GE Healthcare®) and a BioImager were used for signal detection (10 sec exposure).

**References**

1. Martin B, Prudhomme M, Alloing G, Granadel C, Claverys JP (2000) Cross-regulation of competence pheromone production and export in the early control of transformation in *Streptococcus pneumoniae*. Mol Microbiol 38: 867-878.

2. Bergé M, Moscoso M, Prudhomme M, Martin B, Claverys JP (2002) Uptake of transforming DNA in Gram-positive bacteria: a view from *Streptococcus pneumoniae*. Mol Microbiol 45: 411-421.

3. Quevillon-Cheruel S, Campo N, Mirouze N, Mortier-Barrière I, Brooks MA et al. (2012) Structure-function analysis of pneumococcal DprA reveals that dimerization is crucial for loading RecA onto DNA during transformation. Proc Natl Acad Sci USA.

4. Prudhomme M, Camilli A, Claverys JP (2007) in: The Molecular Biology of Streptococci, Hakenbeck R, Chhatwal GS, editors. Horizon Scientific Press, Norfolk, UK, pp. 511-518.

5. Gonzalez A, Llull D, Morales M, Garcia P, Garcia E (2008) Mutations in the *tacF* gene of clinical strains and laboratory transformants of *Streptococcus pneumoniae*: impact on choline auxotrophy and growth rate. J Bacteriol 190: 4129-4138.

6. Chan PF, O'Dwyer KM, Palmer LM, Ambrad JD, Ingraham KA et al. (2003) Characterization of a novel fucose-regulated promoter (PfcsK) suitable for gene essentiality and antibacterial mode-of-action studies in *Streptococcus pneumoniae*. J Bacteriol 185: 2051-2058.

7. Guiral S, Hénard V, Laaberki M-H, Granadel C, Prudhomme M et al. (2006) Construction and evaluation of a chromosomal expression platform (CEP) for ectopic, maltose-driven gene expression in *Streptococcus pneumoniae*. Microbiology (Special Issue on Pneumococcus) 152: 343-349.

8. Pearce BJ, Iannelli F, Pozzi G (2002) Construction of new unencapsulated (rough) strains of *Streptococcus pneumoniae*. Res Microbiol 153: 243-247.

9. Akerley BJ, Rubin EJ, Camilli A, Lampe DJ, Robertson HM et al. (1998) Systematic identification of essential genes by *in vitro mariner* mutagenesis. Proc Natl Acad Sci USA 95: 8927-8932.

10. Martin B, Granadel C, Campo N, Hénard V, Prudhomme M et al. (2010) Expression and maintenance of ComD-ComE, the two-component signal-transduction system that controls X-state (competence) of *Streptococcus pneumoniae*. Mol Microbiol 75: 1513-1528.

11. Prudhomme M, Claverys JP (2007) in: The Molecular Biology of Streptococci, Hakenbeck R, Chhatwal GS, editors. Horizon Scientific Press, Norfolk, UK, pp. 519-524.

12. Zeghouf M, Li J, Butland G, Borkowska A, Canadien V et al. (2004) Sequential Peptide Affinity (SPA) system for the identification of mammalian and bacterial protein complexes. J Proteome Res 3: 463-468.

13. Guiral S, Mitchell TJ, Martin B, Claverys JP (2005) Competence-programmed predation of noncompetent cells in the human pathogen *Streptococcus pneumoniae*: genetic requirements. Proc Natl Acad Sci USA 102: 8710-8715.

14. Donati C, Hiller NL, Tettelin H, Muzzi A, Croucher NJ et al. (2010) Structure and dynamics of the pan-genome of *Streptococcus pneumoniae* and closely related species. Genome Biol 11: R107.

15. Hiller NL, Janto B, Hogg JS, Boissy R, Yu S et al. (2007) Comparative genomic analyses of seventeen *Streptococcus pneumoniae* strains: insights into the pneumococcal supragenome. J Bacteriol 189: 8186-8195.

16. Hiller NL, Eutsey RA, Powell E, Earl JP, Janto B et al. (2011) Differences in genotype and virulence among four multidrug-resistant *Streptococcus pneumoniae* isolates belonging to the PMEN1 clone. PLoS ONE 6: e28850.

17. Feng J, Lupien A, Gingras H, Wasserscheid J, Dewar K et al. (2009) Genome sequencing of linezolid-resistant *Streptococcus pneumoniae* mutants reveals novel mechanisms of resistance. Genome Res 19: 1214-1223.

18. Ding F, Tang P, Hsu MH, Cui P, Hu S et al. (2009) Genome evolution driven by host adaptations results in a more virulent and antimicrobial-resistant *Streptococcus pneumoniae* serotype 14. BMC Genomics 10: 158.

19. Polissi A, Pontiggia A, Feger G, Altieri M, Mottl H et al. (1998) Large-scale identification of virulence genes from *Streptococcus pneumoniae*. Infect Immun 66: 5620-5629.

20. Cerritelli S, Springhorn SS, Lacks SA (1989) DpnA, a methylase for single-strand DNA in the *Dpn* II restriction system, and its biological function. Proc Natl Acad Sci USA 86: 9223-9227.

21. Sung CK, Li H, Claverys JP, Morrison DA (2001) An *rpsL* Cassette, Janus, for Gene Replacement through Negative Selection in *Streptococcus pneumoniae*. Appl Environ Microbiol 67: 5190-5196.
